# Characterization of genetically defined sporadic and hereditary type 1 papillary renal cell carcinoma cell lines

**DOI:** 10.1002/gcc.22940

**Published:** 2021-03-10

**Authors:** Youfeng Yang, Christopher J. Ricketts, Cathy D. Vocke, J. Keith Killian, Hesed M. Padilla‐Nash, Martin Lang, Darmood Wei, Young H. Lee, Darawalee Wangsa, Carole Sourbier, Paul S. Meltzer, Thomas Ried, Maria J. Merino, Adam R. Metwalli, Mark W. Ball, Ramaprasad Srinivasan, W. Marston Linehan

**Affiliations:** ^1^ Urologic Oncology Branch, Center for Cancer Research National Cancer Institute, National Institutes of Health Bethesda Maryland USA; ^2^ Genetics Branch, Center for Cancer Research National Cancer Institute, National Institutes of Health Bethesda Maryland USA; ^3^ Laboratory of Pathology National Cancer Institute, National Institutes of Health Bethesda Maryland USA; ^4^ Present address: Foundation Medicine, Inc Cambridge Massachusetts USA; ^5^ Present address: Division of Urology, Department of Surgery Howard University College of Medicine Washington District of Columbia USA

**Keywords:** CDKN2A, cell line, hereditary type 1 papillary renal cell carcinoma, MET, papillary renal cell carcinoma, pRCC

## Abstract

Renal cell carcinoma (RCC) is not a single disease but is made up of several different histologically defined subtypes that are associated with distinct genetic alterations which require subtype specific management and treatment. Papillary renal cell carcinoma (pRCC) is the second most common subtype after conventional/clear cell RCC (ccRCC), representing ~20% of cases, and is subcategorized into type 1 and type 2 pRCC. It is important for preclinical studies to have cell lines that accurately represent each specific RCC subtype. This study characterizes seven cell lines derived from both primary and metastatic sites of type 1 pRCC, including the first cell line derived from a hereditary papillary renal carcinoma (HPRC)‐associated tumor. Complete or partial gain of chromosome 7 was observed in all cell lines and other common gains of chromosomes 16, 17, or 20 were seen in several cell lines. Activating mutations of *MET* were present in three cell lines that all demonstrated increased MET phosphorylation in response to HGF and abrogation of MET phosphorylation in response to MET inhibitors. *CDKN2A* loss due to mutation or gene deletion, associated with poor outcomes in type 1 pRCC patients, was observed in all cell line models. Six cell lines formed tumor xenografts in athymic nude mice and thus provide in vivo models of type 1 pRCC. These type 1 pRCC cell lines provide a comprehensive representation of the genetic alterations associated with pRCC that will give insight into the biology of this disease and be ideal preclinical models for therapeutic studies.

## 1 INTRODUCTION

Renal cell carcinoma (RCC) does not exist as a single disease but is made up of multiple histologically defined disease subtypes that are each associated with distinctive genetic and genomic alterations. These differences mean that each subtype may require specific management and treatment. Papillary renal cell carcinoma (pRCC) is the second most common subtype of RCC after conventional or clear cell renal cell carcinoma (ccRCC), accounting for ~20% of cases. Papillary RCC is further subcategorized into type 1 and type 2 pRCC. It is important for preclinical studies that cell line models which accurately represent each specific RCC subtype are produced and characterized.

RCC, also referred to as kidney cancer, can occur as either a sporadic or inherited disease, and studies of familial kidney cancer syndromes have elucidated important genetic features of RCC. Investigations of the inherited von Hippel‐Lindau (VHL) clear cell RCC (ccRCC) predisposition syndrome led to the discovery of the *VHL* tumor suppressor gene and loss of function of this gene in VHL renal tumors, as well as in a significant percentage of tumors from patients with sporadic ccRCC (ranging from 59% to 92%).^1^, ^2^, ^3^, ^4^, ^5^ The knowledge gained about the function of the VHL protein product in controlling oxygen sensing and the cells response to hypoxia and the effect of VHL loss on cellular biology has led to the development of targeted therapies.^6^, ^7^, ^8^, ^9^ Similarly, studies of the hereditary pRCC syndromes identified germline activating mutations within the *MET* oncogene in hereditary papillary renal carcinoma (HPRC), associated with type 1 pRCC, as well as germline inactivating mutations in the *FH* tumor suppressor gene in hereditary leiomyomatosis and renal cell carcinoma (HLRCC), often associated with an aggressive variant of type 2 pRCC.^10^, ^11^, ^12^

Alongside these familial studies, analysis of large cohorts of sporadic pRCC, such as those performed by the Cancer Genome Atlas (TCGA), have provided further insights into the biology of these tumors that need to be reflected in cell line models.^13^ Altered chromosomal copy number patterns represent the most common somatic changes in sporadic type 1 pRCC with gains of chromosome 7 (encoding the *MET* gene) and 17 being most frequent, followed by gain of chromosomes 12, 16, and 20.^13^, ^14^, ^15^ In addition to gain, somatic mutation of the *MET* gene occurs in approximately 13% of sporadic type 1 pRCC.^10^, ^16^, ^17^ TCGA analysis of sporadic type 1 pRCC identified additional features including mutations in chromatin remodeling genes (eg, *BAP1*) and that loss of *CDKN2A* (p16) associated with poorer patient outcome.^13^, ^18^

Cell line models provide an important tool for investigating the importance of these discoveries and a preclinical model for testing novel therapeutic agents that may target specific alterations within these tumors. The NCI has a long history of generating genetically defined cell line models from primary or metastatic kidney tumor tissue excised from patients with either sporadic or germline mutation associated RCCs.^19^, ^20^, ^21^, ^22^ In previous publications we have described two cell lines, (UOK262 and UOK268), derived from HLRCC‐associated type 2 pRCC‐like tumors, that have been used to demonstrate the potential preclinical effectiveness of both proteasome inhibitors and ABL1 inhibitors in targeting these cells adaption to oxidative stress.^23^, ^24^, ^25^, ^26^ Another recent publication highlighted a type 2 pRCC cell line derived from a sporadic tumor that demonstrated a *NF2* mutation that dysregulated the HIPPO pathway and created a targetable therapeutic susceptibility to dasatinib, thereby inhibiting the downstream effects of HIPPO pathway dysregulation.^27^

This study characterizes the genetic and genomic alterations in seven type 1 pRCC cell lines derived from either primary kidney tumors or metastatic material excised from patients, including the first cell line derived from a germline *MET*‐mutated HPRC patient. These cell line models provide examples of *CDKN2A* (p16) loss, *BAP1*, and *KRAS* mutation, and type 1 pRCC‐associated chromosomal copy number alterations that should enable the effective evaluation of precision therapies.

## 2 MATERIALS AND METHODS

### 2.1 Material acquisition

All patients from which procured materials were acquired were evaluated and managed for clinical assessment and surgical resection or systematic therapy at the Hatfield Clinical Research Center, National Institutes of Health (NIH) as patients of the Urologic Oncology Branch of the National Cancer Institute (NCI). All patient recruitment and tissue procurement and use were approved by the Institutional Review Board of the National Cancer Institute on either the NCI‐97‐C‐0147 or NCI‐89‐C‐0086 protocols. All patients had provided written informed consent. Relevant clinical data was acquired where possible for all patients. Peripheral blood samples were obtained when possible for DNA extraction to provide germline DNA for each patient.

### 2.2 Cell line production protocol

All novel cell lines were established from either tumor tissue excised during surgery or from a cell pellet derived from ascites. Spontaneously immortal cell lines were generated using the protocols and techniques previously described.^22^ In brief, the tumor tissue was diced into small pieces (~1‐2 mm^3^) and smeared across a 10 cm tissue culture dish or 75 cm^2^ flask and placed under 5 to 8 mL of DMEM media in sterile conditions. Ascites fluid was centrifuged at 4°C at 1000*g* for 5 minutes to produce a cell pellet which was mixed with a small volume of media (5‐8 mL) and placed in a tissue culture flask under sterile conditions. All cells were initially cultivated in DMEM medium containing 25 mM d‐glucose with 2 mM l‐glutamine (ThermoFisher Scientific Inc, Massachusetts) and supplemented with 10% fetal calf serum (Sigma‐Aldrich, Missouri) and x1 Antibiotic‐Antimycotic solution (ThermoFisher Scientific Inc). Cell lines were propagated for over 20 passages to demonstrate immortalization with a passage being performed every 2 to 5 days. Once established, the Antibiotic‐Antimycotic solution was removed from the media to demonstrate that no infection was present within the cell line.

### 2.3 Short tandem repeat analysis and mycoplasma testing

All cell lines were evaluated by short tandem repeat (STR) analysis (Genetica Cell Line Testing, North Carolina) to provide a distinct genetic fingerprint for each line (Table S1). When possible, this was compared with the STR analysis of any normal or tumor tissue derived DNA available from the originating patient. In addition, all cell lines were evaluated for mycoplasma contamination and shown to be negative (Genetica Cell Line Testing).

### 2.4 Spectral karyotyping and FISH analysis

Metaphase spreads were prepared for each cell line and spreads were subsequently hybridized with custom chromosome‐specific fluorescence spectral karyotyping (SKY) probes, imaged, and analyzed using the SkyView software (Applied Spectral Imaging, California, CA), as previously described.^28^ The chromosome complements of 15 to 20 metaphase spreads were analyzed for each cell line, and karyotype descriptions were produced in accordance with the human chromosome nomenclature standards described in ISCN (2013).^29^ A structural aberration or chromosomal gain was considered clonal if two or more metaphase spreads contained the same change, while chromosomal losses were considered clonal if three or more metaphase spreads demonstrated the same loss.^28^, ^29^ A minimum of 15 metaphase spreads were analyzed by fluorescence in situ hybridization (FISH) using probes for two oncogenes *MET* (7q31), *EGFR* (7p11.2), and a probe for the centromere of chromosome 7 (CEP 7). FISH image analysis was performed using the Q‐FISH TM software (Leica).

### 2.5 Nucleic acid extraction

Cell pellets were generated for each cell line from 80‐90% confluent 10 cm dishes and DNAs were extracted using a Promega Maxwell 16 System using the appropriate Maxwell 16 Cell DNA Purification Kit following manufacturers protocol (Promega, Wisconsin). DNAs from the associated patient's blood samples were similarly extracted using a Promega Maxwell 16 Blood DNA Purification Kit following manufacturers protocol (Promega). Total RNA was extracted from each cells line using Trizol Reagent (Invitrogen, California) and following the standard protocol. In brief, cell lines were grown in 10 cm dishes until they reached a confluency of approximately 80% to 90% and then washed once with 5 mL of sterile PBS and lysed using 1 mL of Trizol Reagent. Following the standard Trizol Reagent guanidinium thiocyanate‐phenol‐chloroform RNA extraction protocol, the resulting total RNA was re‐suspended in 50 μL of RNase‐Free water and stored at −80°C. DNA and RNA concentrations and purities were measured using a NanoDrop 2000 UV‐Vis Spectrophotometer (Thermo Fisher Scientific Inc).

### 2.6 Copy number analysis

Copy number analysis was performed using 2 μL of dilute DNA (5 ng/μL), 5 μL of Taqman universal PCR master mix, 0.5 μL of Taqman Copy Number Assay probe (FAM), 0.5 μL of Taqman Copy Number Reference Assay (RNaseP) probe (VIC) and 2.0 μL of RNase‐Free Water to produce a 10 μL reaction volume for analysis by an ABI ViiA7 real‐time PCR system (Thermo Fisher Scientific Inc). Gene copy number was normalized to the Ribonuclease P RNA component H1 (H1RNA) gene (*RPPH1*) on chromosome 14 and calculated using the ViiA7 software as comparative CT (ΔΔCT) values. The nonimmortalized normal kidney cell line, RPTEC, was designated to represent a normal chromosomal copy number with a value of 2. Taqman Copy Number Assay probes (Thermo Fisher Scientific Inc) were used to assess the gene copy number of the following genes: *CDKN2A* (Hs03714372_cn), *MET* (Hs01432482_cn), and *EGFR* (Hs00711969_cn).

### 2.7 Real‐time PCR analysis

cDNA was synthesized from 2 μg of total mRNA from each cell line using the SuperScript VILO cDNA Synthesis Kit (Invitrogen) in a 20 μL volume. The cDNAs were diluted 10‐fold with 180 μL of RNase‐Free Water. RT‐PCR amplification was performed using 2 μL of dilute cDNA, 5 μL of Taqman universal PCR master mix, 0.5 μL of Taqman Gene Expression Assay probe, and 2.5 μL of RNase‐Free Water to produce a 10 μL reaction volume for analysis by an ABI ViiA7 real‐time PCR system (Thermo Fisher Scientific Inc). Expression levels were normalized to the control 18S rRNA gene (Hs99999901_m1) and calculated using the ViiA7 software as comparative CT (ΔΔCT) values. The nonimmortalized normal kidney cell line, RPTEC, was designated to represent the normal expression level with a value of 1. Taqman Gene Expression Assay probes (Thermo Fisher Scientific Inc) were used to assess the expression levels for several genes including, *CDKN2A* (Hs00923894_m1), *MET* (Hs01565584_m1), *EGFR* (Hs01076078_m1), *CD274* (PD‐L1) (Hs00204257_m1), and *PDCD1LG2* (PD‐L2) (Hs00228839_m1).

### 2.8 DNA sequencing and mitochondrial (mt)DNA sequencing

Cell line DNA was sequenced using the OncoVar assay that is a targeted hybrid capture sequencing analysis which detects genomic variants in a panel of 240 cancer‐related genes, including known pRCC associated genes, and was performed as previously published.^30^, ^31^ Mutations identified within the *MET*, *CDKN2A*, *KRAS*, *CUL3*, and *BAP1* genes were validated by PCR using a Qiagen Taq PCR Core Kit (Qiagen, Maryland) followed by bidirectional sequencing using the BigDye Terminator v.1.1 Cycle Sequencing Kit (Applied Biosystems, California), in accordance with the manufacturer's specifications. Validations were compared to a control DNA sample derived from the peripheral blood leukocytes of a control patient. The entire mitochondrial genome was sequenced and analyzed as previously described.^32^ In brief, the entire mitochondrial genome was amplified from whole genomic DNA as overlapping PCR fragments using KAPA2G Fast Readymix (KAPA Biosystems, Massachusetts) and sequenced with BigDye Terminator v.1.1 Cycle Sequencing Kit (Applied Biosystems). All Sequence reactions were cleaned with Performa DTR Plates (Edge Bio, Maryland) and Sanger sequencing was performed on an ABI 3730/ABI 3500xl Genetic Analyzer automated sequencing machine (Applied Biosystems) at the CCR Genomics Core at the National Cancer Institute, NIH, Bethesda, MD 20892. Forward and reverse sequences were evaluated using Sequencher 5.4.6 (Genecodes, Michigan).

### 2.9 Two site immunoassay analysis of MET protein and phosphoprotein

Analysis of the total and phospho‐MET levels in Triton X‐100 cell line extracts was performed by an electrochemiluminescent two site immunoassay using a SectorImager 2400 plate reader (Meso Scale Discovery, Gaithersburg, Maryland) as previously described.^33^ The assay has attomole sensitivity for total MET; pMET was expressed as signal intensity (SI) normalized to MET/total protein. Two MET inhibitors were used INC280 (Capmatinib; INCB28060), a potent, orally active, selective, and ATP competitive c‐Met kinase inhibitor, and EMD1214063 (Tepotinib), a potent and selective c‐Met inhibitor with IC50 of 4 nM, >200‐fold selective for c‐Met than IRAK4, TrkA, Axl, and IRAK1. Cell lines were treated for 20 minutes with either 5 nM INC280 or 5 nM EMD1214063 followed by concurrent treatment with 1 nM HGF for an additional 20 minutes. Treatment of HGF alone were performed for 20 minutes with 1 nM HGF.

### 2.10 Mouse xenograft protocol

Approximately 1 × 10^6^ of cells were suspended in a 0.2 mL mixture of 50% PBS and 50% Matrigel Matrix (Corning Life Sciences, Massachusetts) and subcutaneously injected into the flanks of 5 NCI Athymic NCr‐nu/nu mice (obtained from Charles River Frederick Research Model Facility) to evaluate the tumorigenic potential. All animal care protocols used had been approved by the Institutional Animal Care and Use Committee (IACUC) and were in accordance with National Cancer Institute guidelines.

## 3 RESULTS

### 3.1 Clinical features of patients and cell line derivation

**FIGURE 1:**
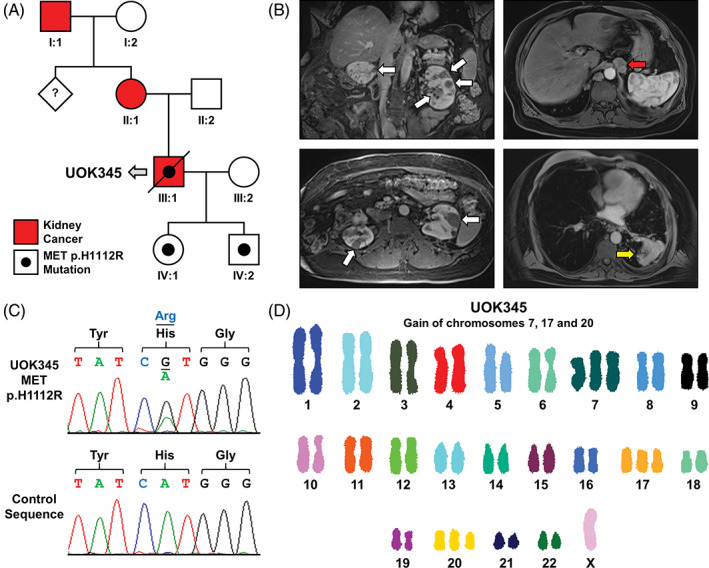
Analysis of the UOK345 cell line model derived from a hereditary papillary renal carcinoma (HPRC) patient. A, Pedigree for the hereditary papillary renal carcinoma family that includes the proband (III:1) from which UOK345 was derived. B, Germline familial mutation in the MET oncogene (p.H1112R) present in the UOK345 cell line. The increased signal peak for the mutant nucleotide is consistent with gain of the mutant allele. C, Coronal and axial CT scans demonstrating multiple, bilateral primary tumors within the kidney (white arrows) and the metastatic spread to local lymph nodes (red arrow) and the lung (green arrow). D, Spectral karyotyping (SKY) analysis of UOK345, showing gain of chromosomes 7, 17, and 20

Seven independent spontaneously immortalized cell lines were derived from material procured from six individuals with sporadic type 1 pRCC and one member from a family with HPRC (Table 1). All seven patients had a histologic diagnosis of type 1 pRCC based on analysis of biopsies or surgical materials (Figure S1). All cell lines were analyzed for their STR genetic fingerprint and matched to original patient normal tissue or tumor DNA where available (Table S1). UOK208, UOK274, UOK342, and UOK345 all matched their respective patient normal tissue STR profiles and UOK112, UOK332, and UOK345 matched their respective tumor DNA STR profiles (Table S1). UOK112 and UOK342 represent the only previously published cell lines from this cohort.^19^, ^27^ UOK112 was derived from a 67‐year‐old male patient who presented with a right‐sided 10.5 cm renal mass and multiple metastatic lung nodules and underwent a radical nephrectomy to remove the kidney tumor that generated the cell line. UOK208 was made from a 64‐year‐old male patient who presented with a left‐sided 8.0 cm renal mass and a metastatic neck mass that demonstrated type 1 pRCC and clear cell RCC features. The renal mass was excised by radical nephrectomy, that also demonstrated positive local lymph nodes, and used to generate a cell line. UOK274 originated from primary tumor from a 53‐year‐old male patient who presented with a left‐sided 13.0 cm renal mass and multiple metastatic lung nodules. UOK332, UOK337, and UOK342 were derived from ascites fluids obtained from three patients with metastatic type 1 pRCC with primary renal masses measuring 10.0, 8.0, and 3.2 cm, respectively. UOK345 was derived from a male patient who initially presented at age 41 and whose mother and maternal grandfather also had kidney cancer (Figure 1A). Germline analysis of the HPRC‐associated *MET* oncogene identified a known pathogenic missense mutation, p.H1112R, thus confirming his diagnosis of HPRC (Figure 1B).^16^ The patient presented with multifocal, bilateral kidney tumors (15 were surgically excised ranging from 0.7 to 3.0 cm) and metastasis to both local lymph nodes and the lung (Figure 1C); he was managed for several years before the UOK345 cell line was generated from a sample of ascites fluid.

**TABLE 1:** Clinical features of patients from whom cell lines were derived

UOB cell line	Gender	Age at diagnosis	Size of primary tumor	Tissue source for cell line derivation
UOK112	Male	67	10.5 cm	Right kidney tumor
UOK208	Male	64	8.0 cm	Lymph node met
UOK274	Male	53	13.0 cm	Left kidney tumor
UOK332	Male	45	10.0 cm	Peritoneal fluid
UOK337	Male	61	8.0 cm	Abdominal fluid
UOK342	Male	63	3.2 cm	Ascites
UOK345	Male	56	Largest tumor 4.0 cm	Pleural fluid

### 3.2 Spectral karyotyping of type 1 pRCC cell lines

**FIGURE 2:**
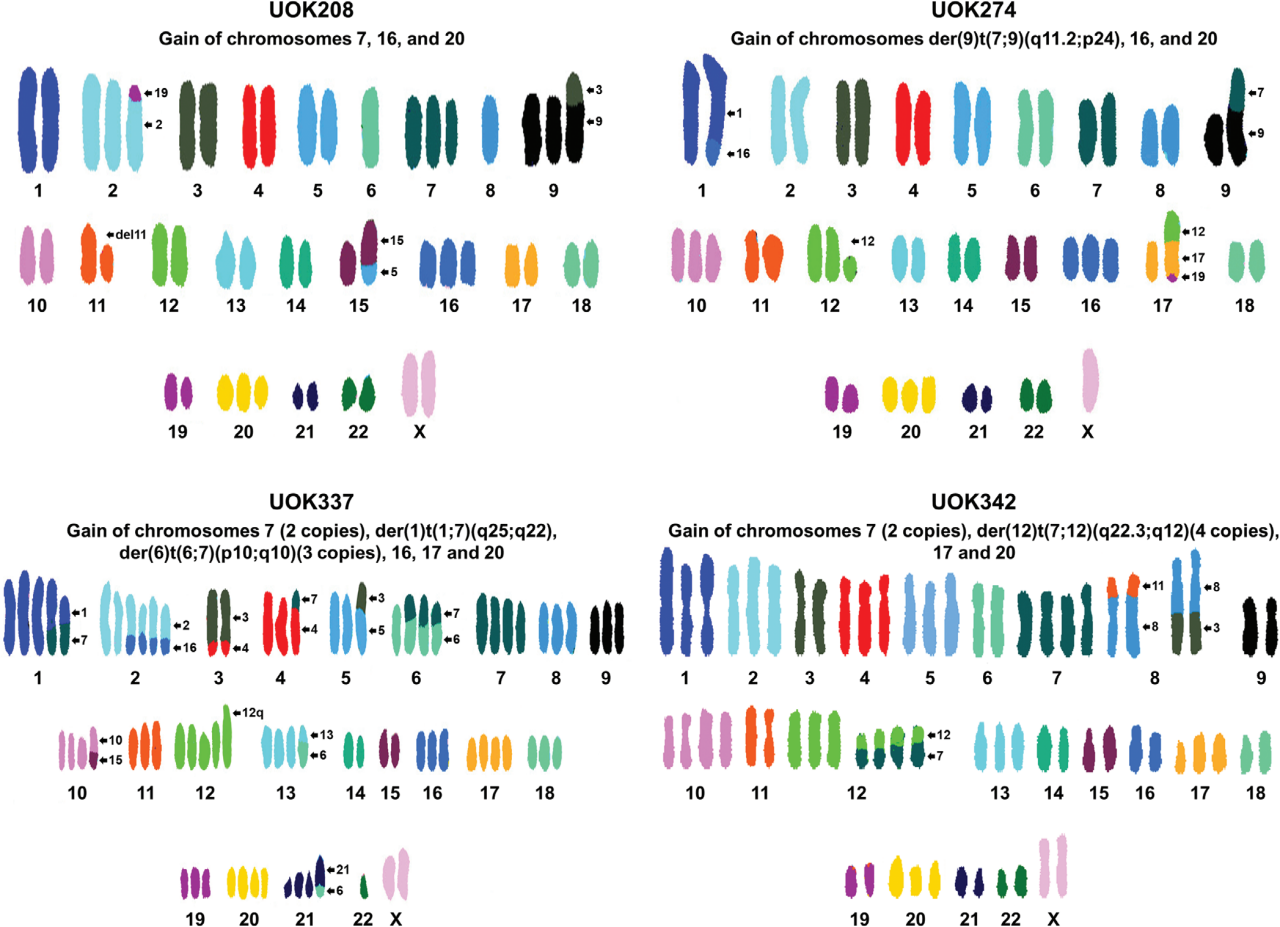
Spectral karyotyping (SKY) analysis of type 1 pRCC cell line models. SKY analyses of UOK208, UOK274, UOK337, and UOK342. UOK208 and UOK274 show a mostly diploid karyotype, while UOK342 had a near triploid chromosomal count and UOK337 had a greater than triploid compliment of chromosomes with many chromosomal translocations. Major chromosomal changes, such as chromosome 7, 16, 17, and 20 gain, are highlighted

Spectral karyotyping demonstrated that five of the seven lines were near diploid exhibiting few copy number chromosomal gains or losses and few translocations (Figures 1D, 2 , and S2; Table 2). UOK342 is a hypo‐triploid (>3n) cell line, and UOK337 is a hyper‐triploid cell line with 10 different unbalanced chromosomal translocations, some duplicated, others only observed as one copy (Figures 1D, 2 , and S2). Whole copy number gains of chromosome 7 were observed in all cell lines, except for UOK274 which only contained an unbalanced translocation that resulted in partial gain of chromosome arm 7q (7q11.2‐>7qter), a region that encodes the *MET* (7q31.2), *BRAF* (7q34), and *HGF* (7q21.11) genes, but not *EFGR* (7p11.2) gene (Figures 1D, 2 , and S2; Table 2). Three cell lines (UOK208, UOK332, and UOK345) has three copies of chromosome 7, and UOK112 has four copies of chromosome 7. UOK337 has four copies of chromosome 7, and three different unbalanced translocations, containing segments from chromosome arm 7q (7q10‐>7qter). UOK342 has four copies of chromosome 7, and partial gains of 7q, resulting from the presence of three to four copies of an unbalanced translocation, der(12)t(7;12)(q22.3;q12). For two cell lines, UOK342 and UOK345, the SKY analysis was further investigated by FISH analysis that confirmed three copies of the *MET* gene in UOK345 and seven copies of the *MET* gene in UOK342 (Figure S3).

**TABLE 2:** Genetic features of type 1 pRCC cell lines

UOB cell line	UOK112	UOK208	UOK274	UOK332	UOK337	UOK342	UOK345
Chromosomal copy number	Diploid	Diploid	Diploid	Diploid	Hyper‐triploid	Hypo‐triploid	Diploid
Chr. 7 gain	+2	+1	+0^a^	+1	+2^a^	+2^a^	+1
Copies of *MET*	+2	+1	+1	+1	+7	+6	+1
Chr. 16 gain	+1	+1	+1		+1		
Chr. 17 gain					+2	+1	+1
Chr. 20 gain		+1	+1	+1	+2	+1	+1
*CDKN2A* (p16) loss	+/+	−/−	−/+	−/+	−/−	−/−	−/−
*CDKN2A* (p16) expression	+++	−	−	−	−	−	−
*MET* mutation	No	p.V1088A (somatic)	No	No	p.H1106Q (somatic)	No	p.H1112R (germline)
Additional mutations	*CDKN2A*		*KRAS*	*BAP1*		*NF2, KRAS*	*CUL3*
Xenograft growth in mice	No	Slow	Slow	Rapid	Rapid	Rapid	Slow

a Additional partial gain(s) of Chr. 7 including *MET* gene; −/−, complete loss of *CDKN2A*; −/+, partial loss of *CDKN2A*.

Chromosome 17 and 16 are frequently gained in type 1 pRCC, but among our cell lines, only three consistently (>50% of spreads) gained whole copy number gains of chromosome 17 (UOK337, UOK342, and UOK345) and four gained copies of chromosome 16 (UOK112, UOK208, UOK274, and UOK337) (Figures 1D, 2 , and S2).^18^ Notably, SKY analysis revealed that six out of the seven cell lines (85.7%) gained complete copies of chromosome 20, however, this was reported as a less frequent event in type 1 pRCC (36.1%) by the TCGA. Although all cell lines were derived from male patients, no cell lines retained their Y chromosomes and 4 lines (UOK208, UOK332, UOK337, and UOK342) have duplications of their X chromosomes. Chromosomal alterations associated with clear cell RCC, such as loss of chromosomes 3p and 14q, or partial gains of chromosome 5q, are not characteristic of our cell lines, and no cell lines demonstrated large‐scale loss of chromosome 9, which contains the tumor suppressor *CDKN2A* (p16).

### 3.3 Targeted mutation analysis of type 1 pRCC cell lines

**FIGURE 3:**
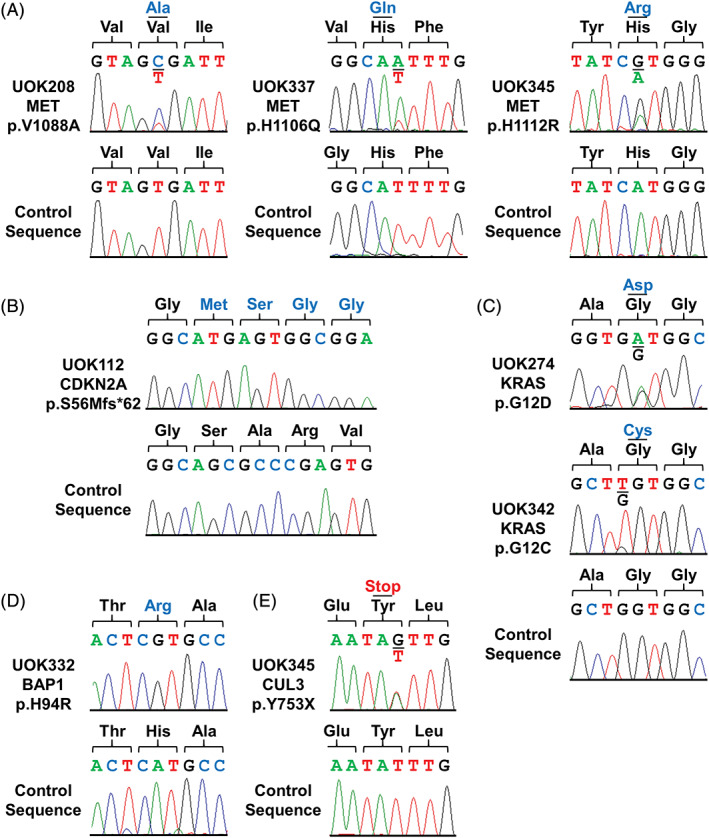
Mutation analysis of type 1 pRCC cell line models. A, Known pathogenic *MET* mutations were seen in UOK208 and UOK345, and a novel *MET* mutation was observed in UOK337. B, A homozygous truncating mutation of *CDKN2A* that encodes p16 in UOK112. C, Known pathogenic activating mutations of *KRAS* in UOK274 and UOK342. D, A homozygous missense mutation of *BAP1* in UOK332. E, A heterozygous nonsense mutation of *CUL3* in UOK345

Potential driver mutations within the cell lines were identified utilizing a targeted sequencing assay, OncoVar v3, which includes approximately 240 known cancer genes (Table 2). In addition to the germline *MET* p.H1112R mutation present in UOK345, UOK208 was found to have a known pathogenic *MET* mutation, p.V1088A, and UOK337 demonstrated a novel missense mutation, p.H1106Q. All of these mutations occur in the MET tyrosine kinase domain.^17^ In all three cell lines, confirmatory Sanger sequencing showed increased signal for the mutant allele over the wild‐type indicating that the gained copies of chromosome 7 contained the mutation (Figure 3A). UOK112 had a homozygous frameshift mutation in the *CDKN2A* tumor suppressor gene, a key component of cell cycle regulation (Figure 3B). UOK274 and UOK342 demonstrated known pathogenic, activating mutations of the *KRAS* oncogene, p.G12D and p.G12C, respectively (Figure 3C). UOK332 had a homozygous missense mutation, p.H94R, in the *BAP1* tumor suppressor gene, a chromatin remodeling gene, while UOK345 had a heterozygous nonsense mutation in the *CUL3* gene, a regulating component of the NRF2 antioxidant response pathway (Figure 3D and E).

In addition to analyzing the nuclear genome, mutation analysis of the mitochondrial genome was also performed due to mutations observed in the TCGA. The TCGA graded mitochondrial alterations into pathogenic and nonpathogenic mutations with pathogenic mutations having either missense/nonsense mutations with high heteroplasmy or frameshift mutations with greater than 50% heteroplasmy (summarized in Figure S4).^18^ UOK345 was the only cell line that demonstrated a pathogenic change within the mitochondrial genome with a homoplasmic missense mutation in ND5 (p.R436H) (Figure S4). Therefore, only 14.3% of the cell lines had mitochondrial mutations, in comparison to 34% seen in all sporadic pRCC in the TCGA.

### 3.4 Focal *CDKN2A* deletion and expression analysis of *CDKN2A*, *MET*, and *EGFR*

**FIGURE 4:**
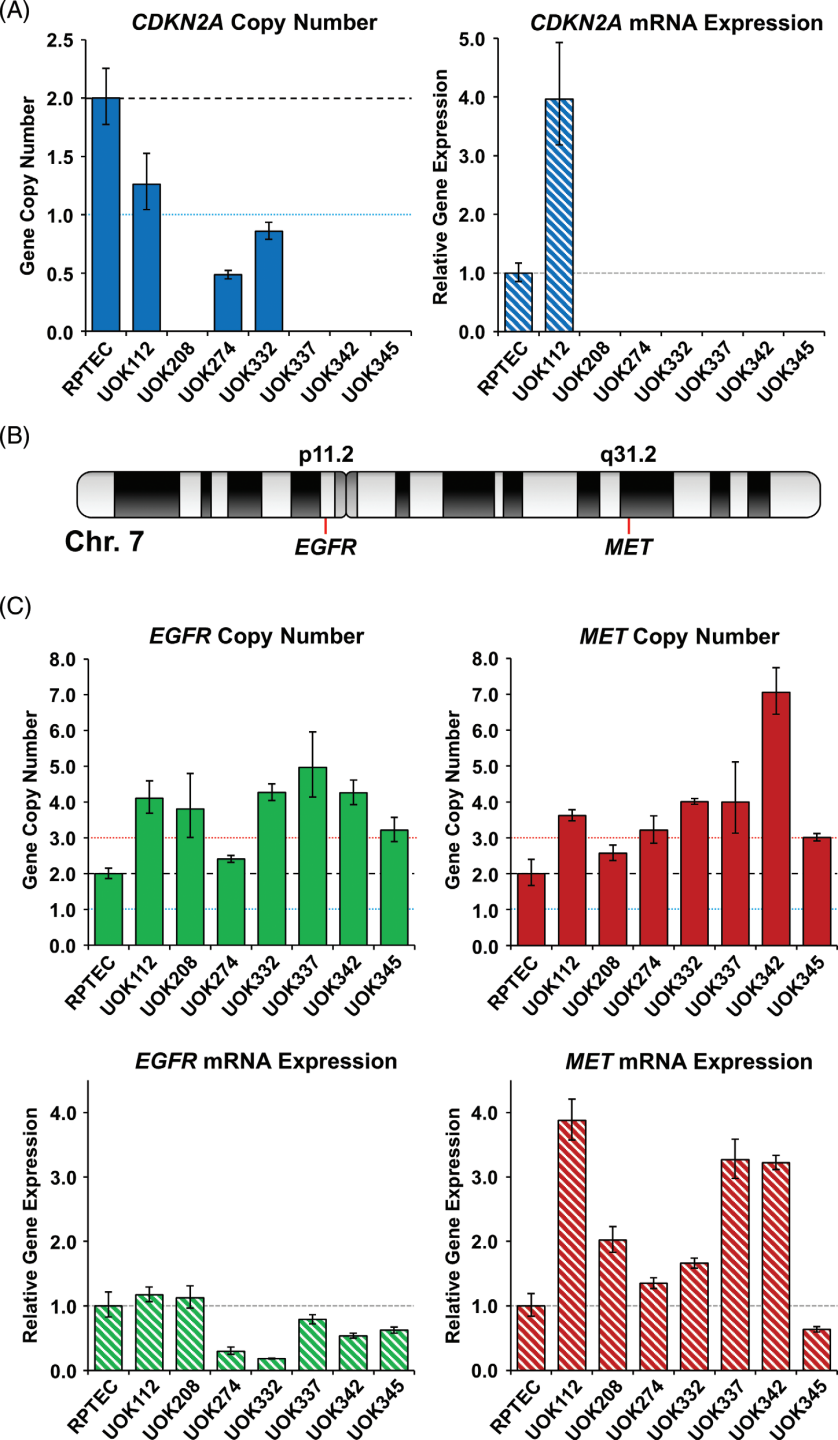
Copy number and mRNA expression analysis of *CDKN2A*, *MET*, and *EGFR*. A, The *CDKN2A* gene copy number and mRNA expression were measured by Taqman Gene Expression Assay probe, Hs00923894_m1, and Taqman Copy Number Assay probe, Hs03714372_cn, and normalized to the RPTEC normal kidney cell line. B, A schematic of chromosome 7 and Taqman copy number analysis of *EGFR* (Hs00711969_cn) and *MET* (Hs01432482_cn) normalized to RPTEC. C, Taqman mRNA expression analysis of *EGFR* (Hs01076078_m1) and *MET* (Hs01565584_m1) normalized to RPTEC

The mutation of *CDKN2A*, the gene that encodes p16, in UOK112 is important as loss of *CDKN2A* has been shown to associate with more aggressive pRCC. However, *CDKN2A* is more frequently lost due to specific gene deletion or promoter methylation.^13^, ^18^ To evaluate the potential for focal gene deletion that would not be detectable by SKY analysis, Taqman‐based gene copy number analysis was performed and demonstrated complete loss of *CDKN2A* in UOK208, UOK337, UOK342, and UOK345. UOK112 had heterozygous loss of *CDKN2A* and both UOK274 and UOK332 demonstrated a high degree of *CDKN2A* copy loss that was not complete, potentially suggesting some degree of heterogeneity within those cell lines (Figure 4A, Table 2). Expression analysis revealed no *CDKN2A* mRNA expression in 6 out of 7 cell lines including UOK274 and UOK332. Expression was only present in the *CDKN2A*‐mutant UOK112 cell line (Figure 4A, Table 2).

Gain of a complete copy of chromosome 7 results in the gain of several potential oncogenes including *MET* at 7p31.2 and *EGFR* at 7p11.2 (Figure 4B) but gain of copy number does not necessarily equate to increased expression of the gene. Taqman‐based analysis of gene copy number confirmed the gain of the *MET* gene in all cell lines and gain of *EGFR* in all cell lines except UOK274 (Figure 4C). However, this results in increased mRNA expression levels of *MET* in all cell lines except UOK345 in comparison to the normal kidney cell line RPTEC, but no increased mRNA expression of the *EGFR* gene in any cell line (Figure 4C).

TCGA analysis had previously shown that the expression levels of PD‐L1 and PD‐L2 are low in type 1 pRCC, in comparison to clear cell RCC that also shows considerable higher levels of immune infiltration.^18^, ^34^ PD‐L1 (encoded by *CD274*) mRNA expression levels were reduced in comparison to the RPTEC normal control in most cell lines and only mildly elevated in UOK112 and comparable to RPTEC in UOK337 (Figure S5), whereas PD‐L2 (encoded by *PDCD1LG2*) mRNA expression levels were similar to RPTEC normal control in most cells and elevated in a single cell line, UOK112 (Figure S5).

### 3.5 Analysis of MET phosphorylation and response to phospho‐MET inhibitors

**FIGURE 5:**
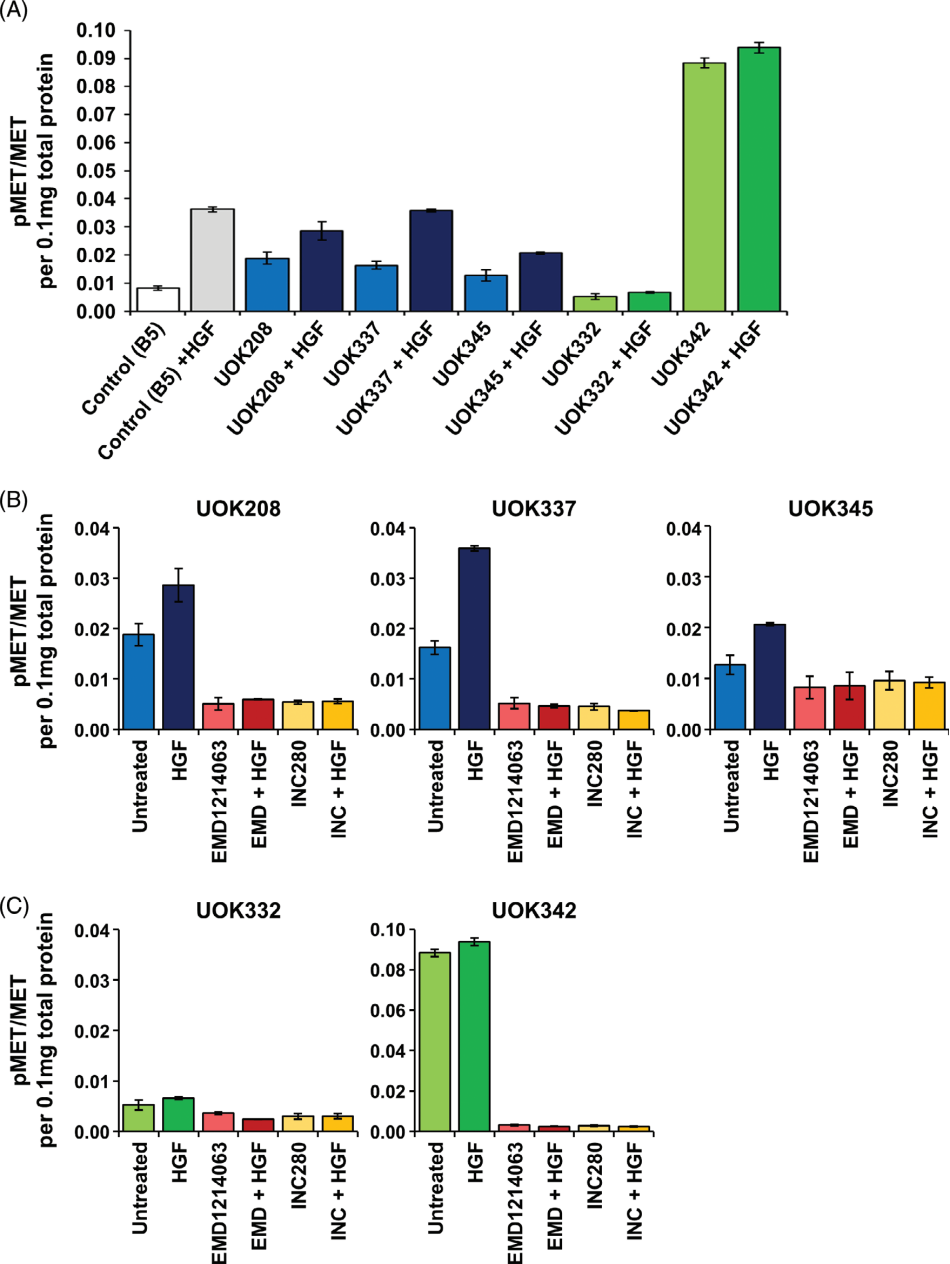
Evaluation of MET inhibitors in pRCC cell line models. A, MSD (Meso Scale Discovery) analysis was used to evaluate the ratio between phosphorylated MET protein and total MET protein (pMET/MET) in the three cell lines with *MET* mutation (UOK208, UOK337, and UOK345) and two cell lines with high *MET* copy number (UOK332 and UOK342) both with and without a 20 minutes exposure to 1 nM hepatocyte growth factor (HGF), the ligand for MET. The breast cancer cell line B5/589 (B5) was used as a positive control for MET expression. B, The response of pRCC cell lines with *MET* mutation to either 5 nM EMD1214063 or 5 nM INC280, both with and without 1 nM HGF. C, The response of pRCC cell lines with high *MET* copy number to either 5 nM EMD1214063 or 5 nM INC280, both with and without 1 nM HGF

MET protein phosphorylation was evaluated in the three cell lines with MET tyrosine kinase domain mutations (UOK208, UOK337, and UOK345), two cell lines with high *MET* copy number (UOK332 and UOK342), and a breast cancer cell line, B5/589 (also known as 184‐B5) that served as a positive control for MET expression. Adding hepatocyte growth factor (HGF), the ligand for the MET receptor, significantly increased phospho‐MET levels in the three cell lines with MET mutations as well as the B5/589 positive control, but the two cell lines with gain of the MET gene showed little response to HGF (Figure 5A). The cell lines were then treated with two different MET inhibitors, EMD1214063 (Tepotinib) and INC280 (Capmatinib). Both EMD1214063 and INC280 similarly reduced the levels of phospho‐MET in all five cell lines to levels lower than those observed in the untreated cells, even in the UOK332 cell line that demonstrated relatively little phospho‐MET (Figure 5B and C). The effect of both MET inhibitors was unaffected by the presence or absence of HGF.

### 3.6 Evaluation of mouse xenograft growth for the type 1 pRCC cell lines

**FIGURE 6:**
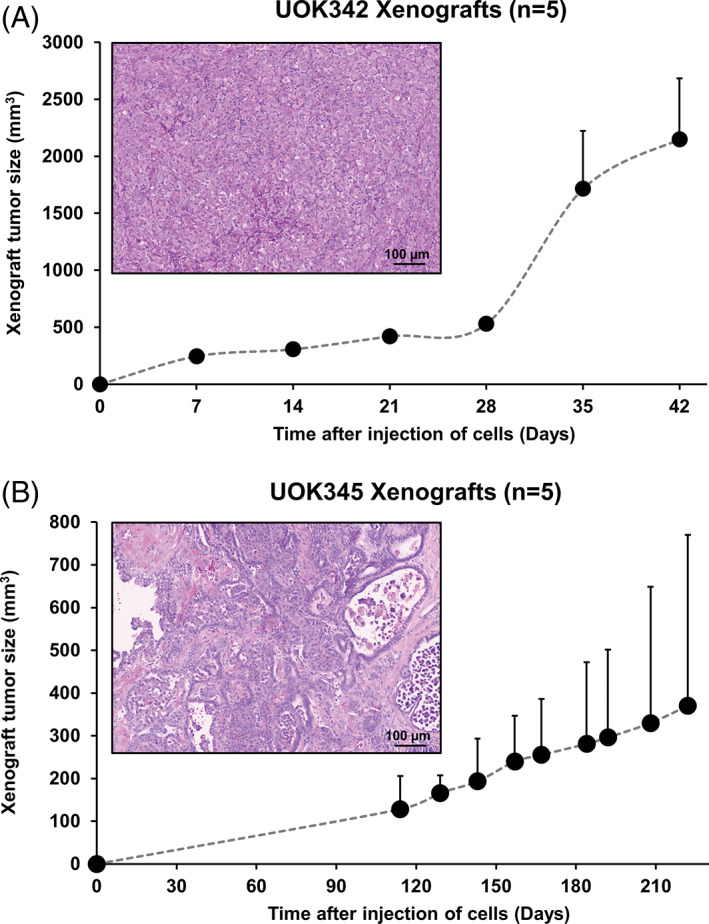
Mouse xenografts of the UOK342 and UOK345 type 1 pRCC cell line models. Five NCI athymic NCr‐nu/nu mice were subcutaneously injected in the flank with approximately 1 million cells of either UOK342 or UOK345 to evaluate the rate of xenograft tumor growth for these type 1 pRCC cell line models. Representative hematoxylin and eosin stained slides from xenografts for each cell line show typical type 1 pRCC histology

All of the type 1 pRCC cell lines, except UOK112, produced xenograft tumors when injected into the flank of athymic nude mice. The cell lines demonstrated a wide variety of growth patterns with UOK345 xenografts growing slowly and taking over 6 months to reach over 300 mm^2^, while UOK342 xenografts grew rapidly producing 2000 mm^2^ tumors within ~40 days (Figure 6, Table 2). UOK208 and UOK274 also produced slow growing xenografts, while UOK332 and UOK337 xenografts grew more rapidly (Figure S6, Table 2). UOK208, UOK332, and UOK345 xenograft tumors demonstrated similar histopathologic patterns to those seen in the original tumors (Figures 6, S1, and S7). UOK274 and UOK337 showed evidence of papillary histology, but no images of the originating tumors were available for comparison (Figure S7). The rapidly growing UOK342 xenografts demonstrated little evidence of papillary structure and looked like solid tumors (Figure 6). Xenografts were analyzed for the presence of key mutations to confirm that they were derived from the appropriate cell line (Figure S8).

## 4 DISCUSSION

The Cancer Genome Atlas (TCGA) analyses of papillary renal cell carcinoma (pRCC) provided an excellent characterization of the major genetic and genomic features of type 1 pRCC.^13^, ^18^ To fully utilize these data, it is important to have cell line models derived from patient tumors that can represent these features, in order to allow further investigation of the biology of type 1 pRCC and evaluation of novel therapeutic agents. This study presents the characterization of several new cell line models for type 1 pRCC, a disease that is currently underrepresented by existing cell line models.

All seven cell line models described herein were spontaneously immortalized from fresh patient tissues, requiring no additional genetic manipulation, and should provide the best representation of type 1 pRCC tumors. Five of the cell line models were derived from metastatic tissues, while the remaining two cell line models were derived from the primary kidney tissue in patients that also had synchronous metastatic disease. Thus, these cell line models may not be appropriate for investigating the biology behind the initiation of type 1 pRCC but rather can provide invaluable insights into the biology of metastatic type 1 pRCC and an excellent preclinical model for novel therapeutics for patients with advanced disease.

Chromosomal alterations are common in RCC and specific gains or losses are associated with different RCC subtypes. Type 1 pRCC is associated with gains of chromosomes 7, 16, and 17, all of which were represented in our type 1 pRCC cell line models. Classic gain of chromosome 7 was observed in 6 out of 7 cell lines. Notably, the UOK274 cell line had a partial gain of chromosome 7q that included *MET*, along with many other genes, but excluded *EGFR*. Similarly, UOK337 and UOK342 had gains of both complete copies of chromosome 7 and additional partial regions of chromosome 7q, leading to more increased copies of *MET* than *EGFR*. The gain of chromosome 7 could potentially affect many oncogenes including both *MET* and *EGFR* but the selective gain of *MET* and the correlated increased in *MET* mRNA expression, that was not observed in *EGFR*, suggests that *MET* gain is a key element in these cell lines.

TCGA and previous studies have shown *MET* to be the most commonly mutated gene in sporadic type 1 pRCC (~15%) and germline MET mutations are associated with HPRC, a disease defined by bilateral, multifocal type 1 pRCCs.^10^, ^13^, ^18^, ^35^ Three of the type 1 pRCC cell line models (UOK208, UOK337, and UOK345) demonstrated activating mutations of the *MET* gene, including the first cell line ever derived from a tumor excised from an HPRC patient (UOK345), and all cell lines demonstrated *MET* gain. This study showed phosphorylation of the mutant MET protein that could be further induced by adding the HGF ligand, and that this phosphorylation‐related activation can be abrogated using existing MET inhibitors, such as the investigation MET Kinase Inhibitor INC280 currently under clinical trial (https://clinicaltrials.gov/ct2/show/NCT02019693). Additionally, cell lines with wild‐type MET gain showed HGF‐independent phosphorylation of MET that could also be abrogated with MET inhibitors. Trials of small molecule inhibitors of MET, including foretinib and savolitinib, have demonstrated some efficacy in pRCC tumors. Foretinib was shown to produce a partial response in 5 out of 10 (50%) of germline MET mutation patients and 1 out of 5 (20%) of somatic MET mutation patients but only 1 out of 18 (5%) of patients with only a gain of chromosome 7.^36^ Subsequently, a study of savolitinib in pRCC patients demonstrated partial response in 18% of participants with MET‐driven disease and none in patients with MET‐independent disease..^37^ Thus, these cell lines models can provide a reliable foundation for the investigation of novel MET inhibitors and evaluating the effect of MET downregulation on the biology of these tumors.

Less frequently observed mutations in pRCC were also present in the new cell line models, with *BAP1* mutated in UOK332 and *CUL3* mutated in UOK345. In TCGA, mutation of the chromatin remodeling gene *BAP1* and the NRF2/ARE (antioxidant responsive element) pathway gene *CUL3* were observed in 5.6% and 4.1% of all pRCC respectively.^18^ Notably, two cell lines (UOK274 and UOK342) had known pathogenic activating mutation of the *KRAS* gene, p.G12D and p.G12C respectively. Pathogenic mutation of *KRAS* was a rare event within the primary pRCC tumors investigated by the TCGA, present in only 2 out of 155 type 1 pRCC (1.3%) and 3 out of 67 type 2 pRCC (4.5%).^13^ Investigation of RAS inhibitors is a very active area of therapeutic research and could provide a therapeutic option for a fraction of type 1 pRCC patients that could be evaluated in these model cell lines.^38^

The presence of these less frequent events in our cell lines may reflect the aggressive nature of the tumors from which they were derived. The TCGA type 1 pRCC samples were largely lower stage tumors and these cell lines were all derived from patients with high stage, metastatic disease. In particular, loss of *CDKN2A* was shown by the TCGA to be associated with poor patient outcome and loss of *CDKN2A* was observed in all of our cell line models.^13^ Understanding the role of *CDKN2A* loss in advanced disease by studying these cell line models could elucidate potential new therapies, such as targeting the cyclin dependent kinases 4 and 6 (CDK4 and CDK6) that are the inhibitor targets of the p16 protein encoded by *CDKN2A*.

Finally, both in vitro and in vivo analysis of cell line models is essential to provide convincing data for the translation of potential new therapies into patients. Six of these cell lines were capable of producing xenograft tumors when injected into the flank of athymic nude mice, providing models for the vast array of genetic alterations that occur within type 1 pRCC and representing both primary and metastatic disease.

It is important to note that there are several models for pRCC currently available that have been characterized to varying degrees, including UOK112 and UOK342 that are characterized in this study. UOK112 was initially published over a decade ago with minimal genetic characterization and UOK342 was first published recently as an example of a pRCC cell line with a somatic NF2 mutation that responds to dasatinib treatment.^19^, ^27^ Other than the NCI derived models, the few commercially available pRCC cell lines are derived from metastatic tissues and lack exact details concerning the primary tumor in the originating patient. A recent study by Sinha et al compared the expression profiles of commercially available RCC cell lines with the expression profiles from over 800 RCC tumors evaluated by the TCGA, including ccRCC, pRCC, and chRCC tumors.^39^ This identified the ACHN, CAL54 and U031 cell lines as clustering within pRCC and all three lines demonstrate gains of chromosome 7 and 17 that are associated with type 1 pRCC.^39^ It is notable that ACHN has been published over time as an example of both a ccRCC cell line and a pRCC cell line but lacks *VHL* mutation, chromosome 3p loss or any other genomic alteration suggestive of ccRCC while demonstrating the gain of chromosomes 7 and 17 associated with pRCC.^40^ The general lack of well‐defined existing models of type 1 pRCC emphasizes the importance the well characterized cell lines in this study.

Thus, these novel type 1 pRCC cell lines significantly increase the number of cell line models currently available for studying type 1 pRCC and provide a comprehensive representation of the genetic alterations associated with this cancer. These cell lines will give valuable insight into the biology of advanced type 1 pRCC tumors and provide an ideal preclinical model to investigate novel therapeutic approaches for pRCC that will hopefully provide the foundation for the development of effective forms of therapy for patients with metastatic disease.

## Supporting information


**Appendix** S1: Supporting Information

## Data Availability

The data that supports the findings of this study are available in the supplementary material of this article or available from the corresponding author upon request.
