# Supplementary material for: Characterization of genetically defined sporadic and hereditary type 1 papillary renal cell carcinoma cell lines
Source: Genes Chromosomes Cancer. 2021 Mar 10;60(6):434–46. doi: 10.1002/gcc.22940 (PMC8251606; doi:10.1002/gcc.22940)
Supplement: Supplementary file 1 — Appendix S1: Supporting Information [file GCC-60-434-s001.docx]

**Supplementary Data**

**for**

**Characterization of genetically defined sporadic and hereditary type 1 papillary renal cell carcinoma cell lines**

Youfeng Yang^1*^, Christopher J. Ricketts^1*^, Cathy D. Vocke^1^, J. Keith Killian^2,#^, Hesed M. Padilla-Nash^2^, Martin Lang^1^, Darmood Wei^1^, Young H. Lee^1^, Darawalee Wangsa^2^, Carole Sourbier^1^, Paul S. Meltzer^2^, Thomas Ried^2^, Maria J. Merino^3^, Adam R. Metwalli^1,†^, Mark W. Ball^1^, Ramaprasad Srinivasan^1^ and W. Marston Linehan^1^

Affiliations:

^1^ Urologic Oncology Branch, Center for Cancer Research, National Cancer Institute, National Institutes of Health, Bethesda, MD, 20892.

^2^ Genetics Branch, Center for Cancer Research, National Cancer Institute, National Institutes of Health, Bethesda, MD 20892.

^3^ Laboratory of Pathology, National Cancer Institute, National Institutes of Health, Bethesda, MD 20892.

^*^ Both authors contributed equally to this manuscript.

^#^ Current address - Foundation Medicine, Inc., Cambridge, MA 02141.

^†^ Current address - Division of Urology, Department of Surgery, Howard University College of Medicine, Washington, DC 20059.


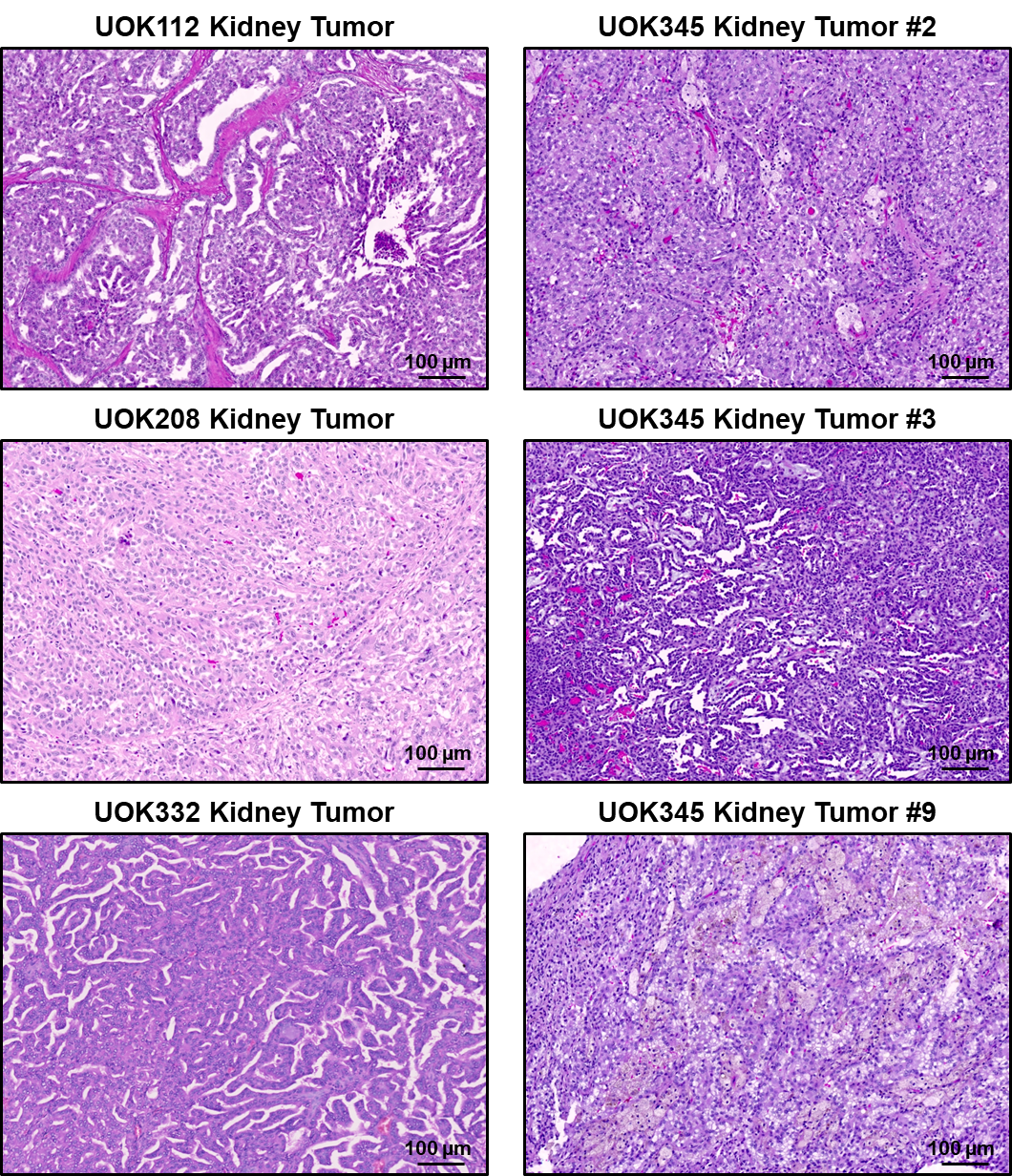


**Figure S1: H&E staining of original type 1 PRCC kidney tumors**

These are representative H&E staining images from the original type 1 PRCC kidney tumors in patients from whom four of the type 1 PRCC cell lines were derived (UOK112, UOK208, UOK332, UOK345). For the HPRC patient from which UOK345 was derived, H&E staining images from three separate tumors are shown.


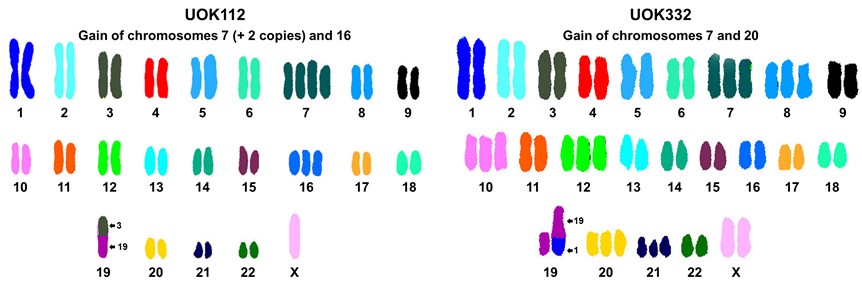

**Figure S2: SKY analysis of UOK112 and UOK332 and summary table of all SKY data**

Representative spectral karyotype (SKY) spreads for UOK112 and UOK332 are shown with the most relevant chromosomal alterations highlighted. The table shows the complete description of the SKY analysis, including chromosomal count/range, for all seven cell lines.


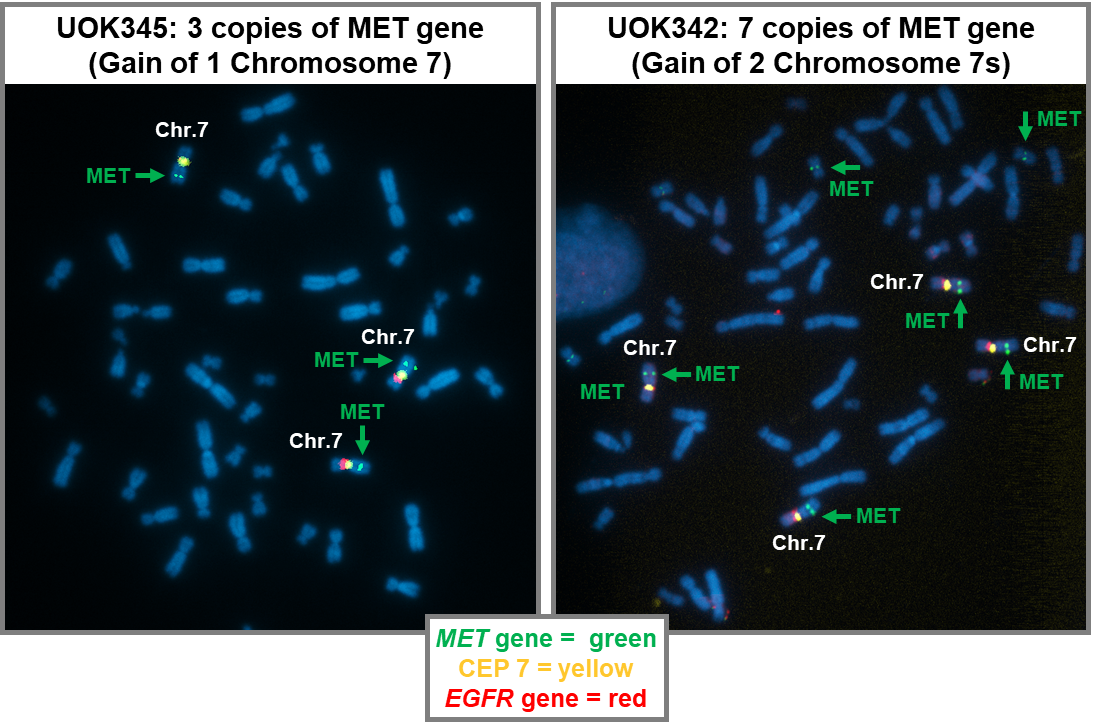


**Figure S3: FISH analysis of *MET* and *EGFR* in UOK342 and UOK345.**

UOK345 and UOK342 type 1 PRCC cell lines were additionally evaluated by (FISH) using a probe for MET (green), EGFR (red) and the CEP 7 centromeric probe for chromosome 7 (yellow).


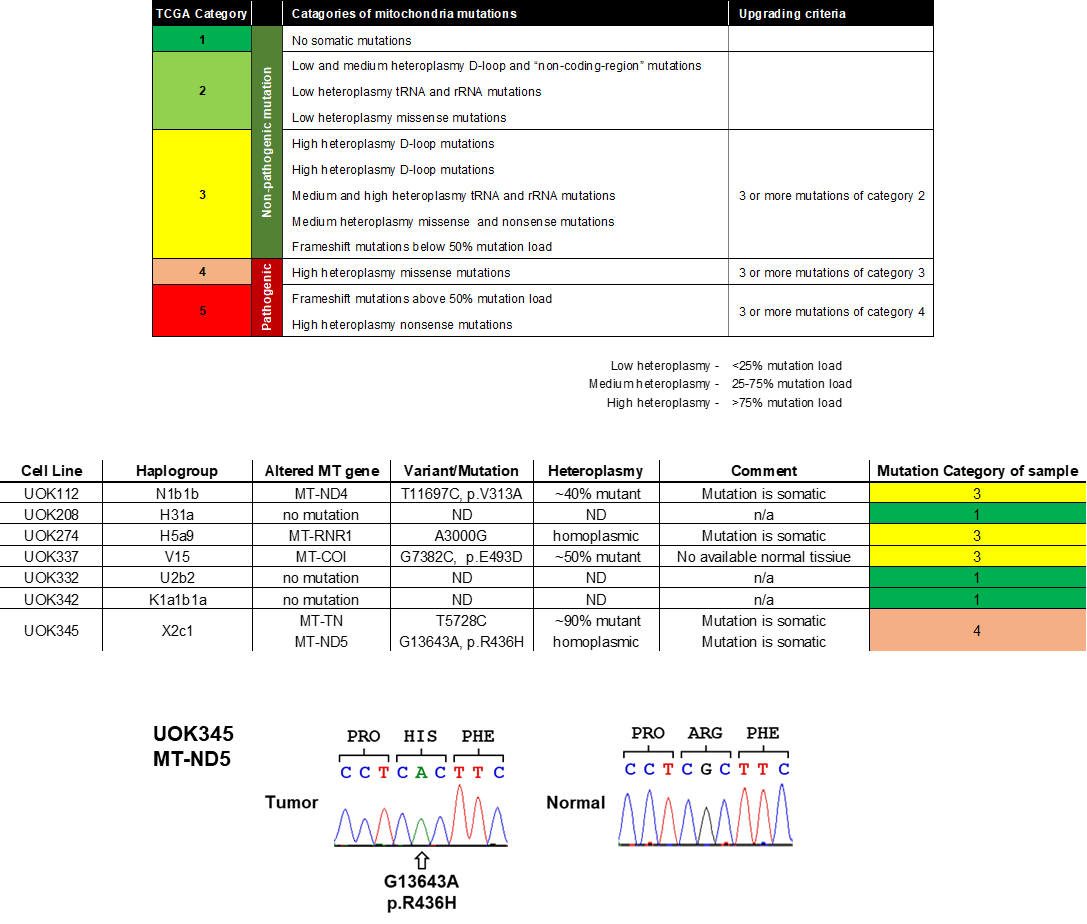


**Figure S4: Analysis of the mitochondrial genome in type 1 PRCC cell lines**

Mitochondrial alterations were evaluated using the same criteria presented in the TCGA analysis of RCC (Upper table). Mitochondrial genome mutations and haplogroup were evaluated for all seven cell lines and mutation were scored using the TCGA mutation category (Lower table). The chromatogram for the MT-ND5 mutation in UOK345 demonstrated a homoplasmic pathogenic missense mutation, p.R436H.


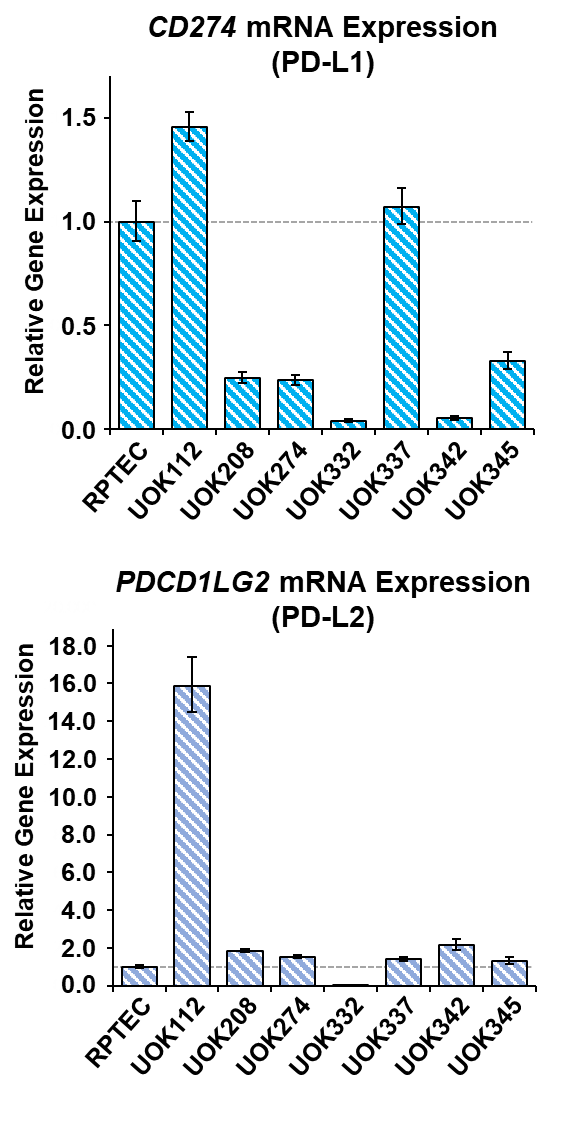


**Figure S5: Expression analysis of PD-L1 and PD-L2 in type 1 pRCC cell lines**

Taqman analysis of mRNA expression of the *CD274* gene that encodes PD-L1 and *PDCD1LG2* the gene that encodes PD-L2 in type 1 pRCC cell lines. Expression levels were compared to the RPTEC normal kidney epithelial cell line (relative expression value of 1.0) and normalized to 18S rRNA expression.


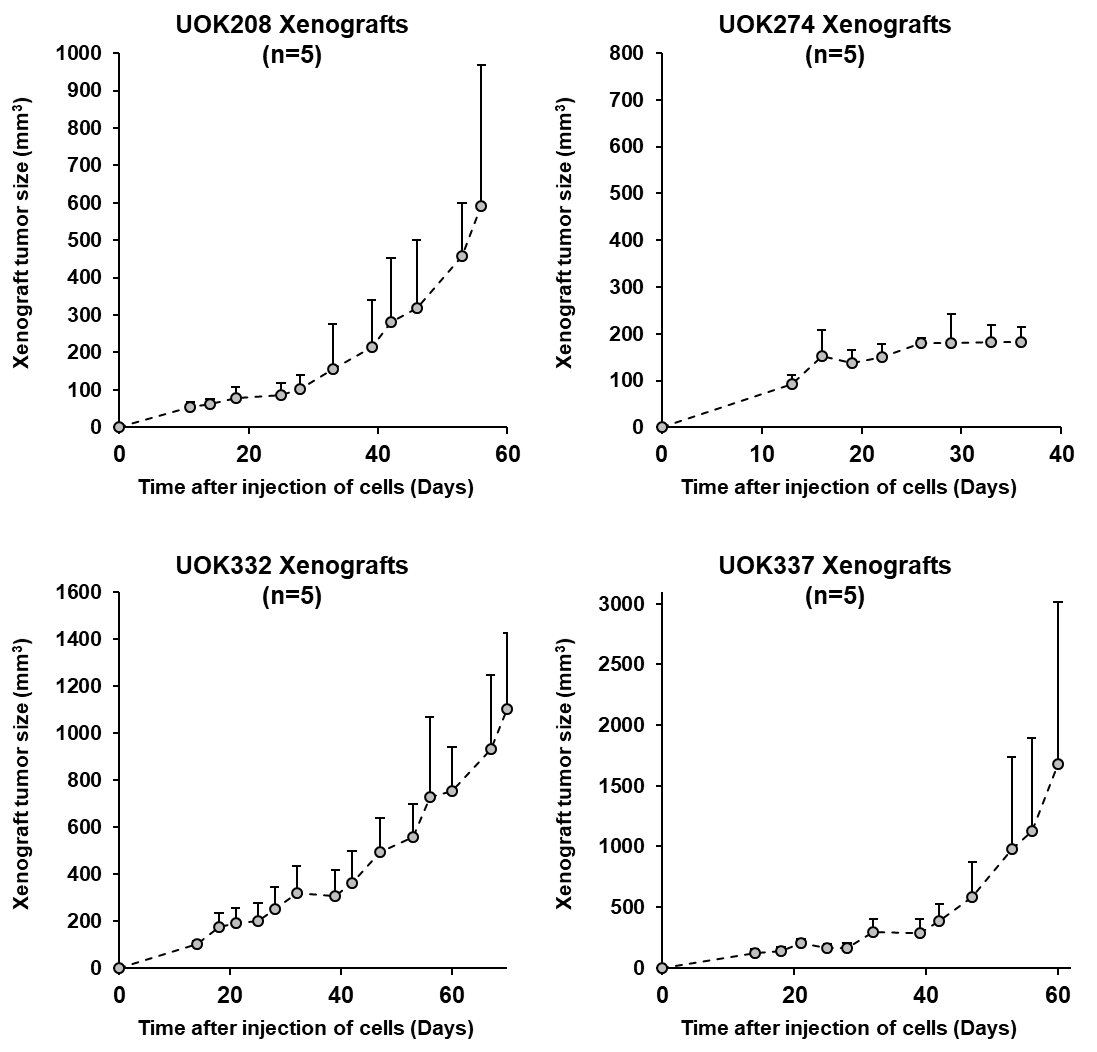


**Figure S6: Growth curves for type 1 PRCC cell line derived xenograft tumors**

For each cell line, five NCI athymic NCr-nu/nu mice were subcutaneously injected in the flank with approximately 1 million cells and growth for approximately 2 months to evaluate the rate of xenograft tumor growth for these type 1 pRCC cell line models.


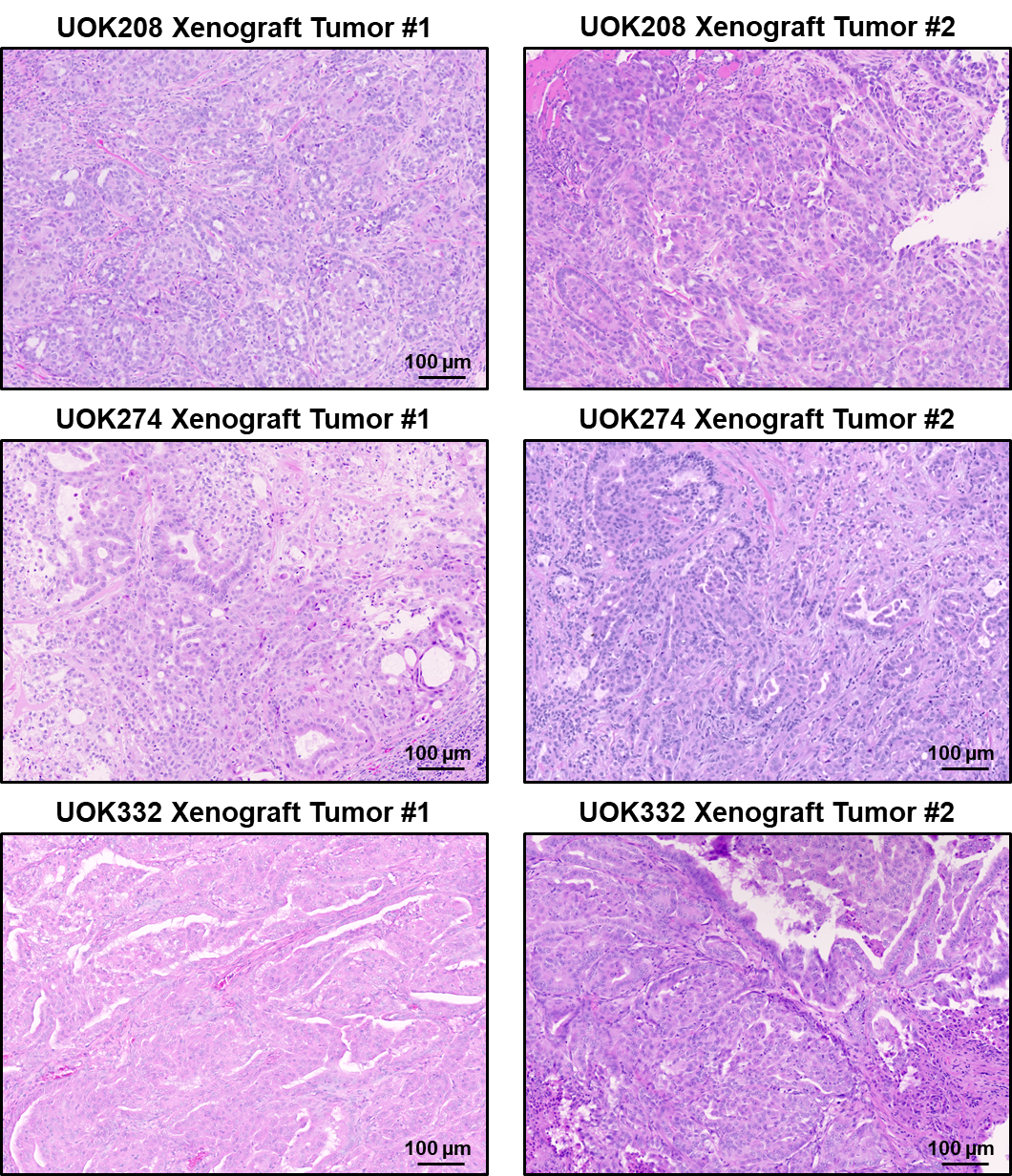


**Figure S7: H&E staining of type 1 PRCC cell line derived xenograft tumors**

These are representative H&E staining images from the six type 1 PRCC cell lines that produced xenograft tumors in the flanks of nude mice. They demonstrate evidence of the type 1 PRCC histologic features observed in the original tumors from which the cell lines were created.


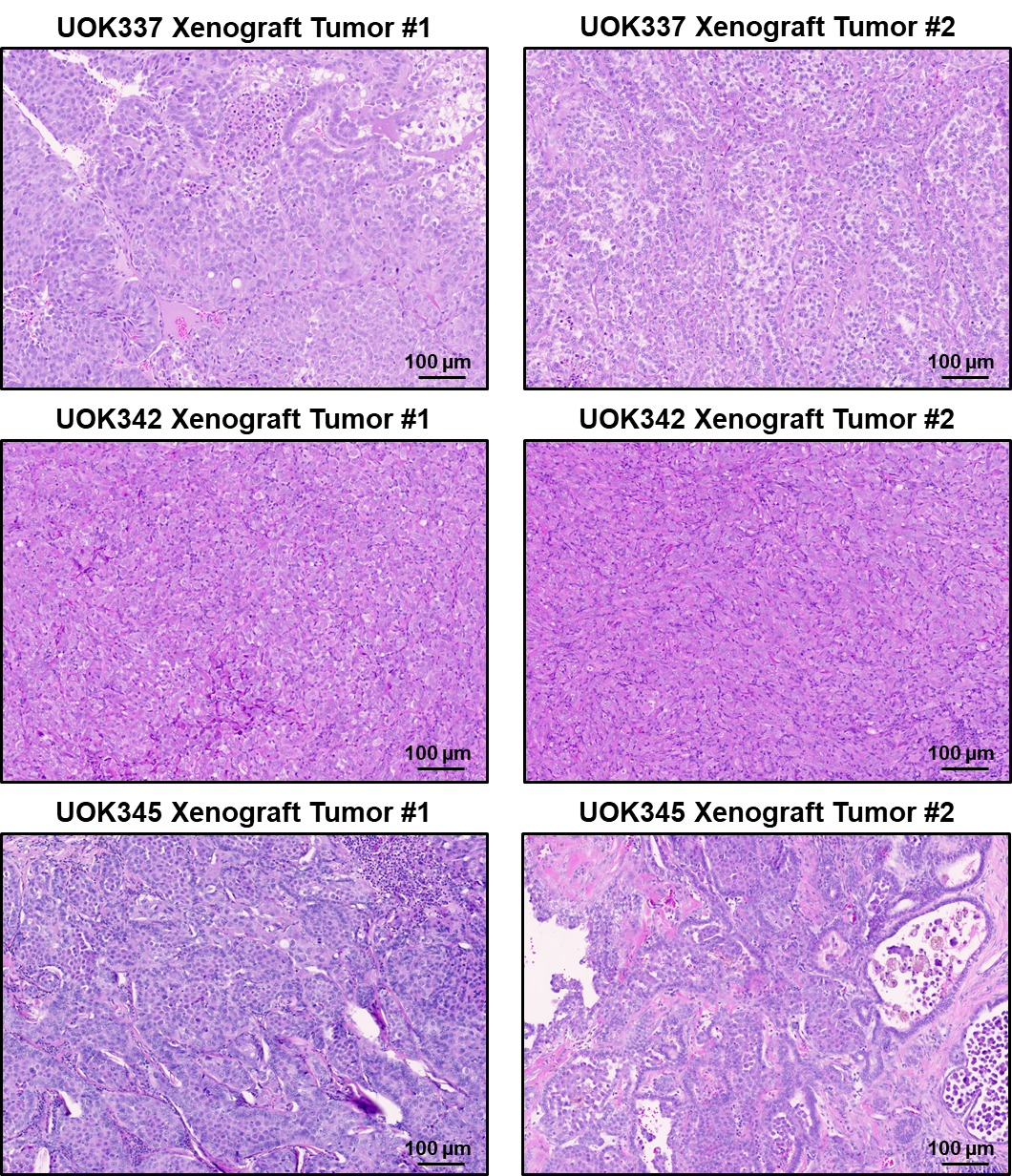


**Figure S7: H&E staining of type 1 PRCC cell line derived xenograft tumors (cont..)**

These are representative H&E staining images from the six type 1 PRCC cell lines that produced xenograft tumors in the flanks of nude mice. They demonstrate evidence of the type 1 PRCC histologic features observed in the original tumors from which the cell lines were created.


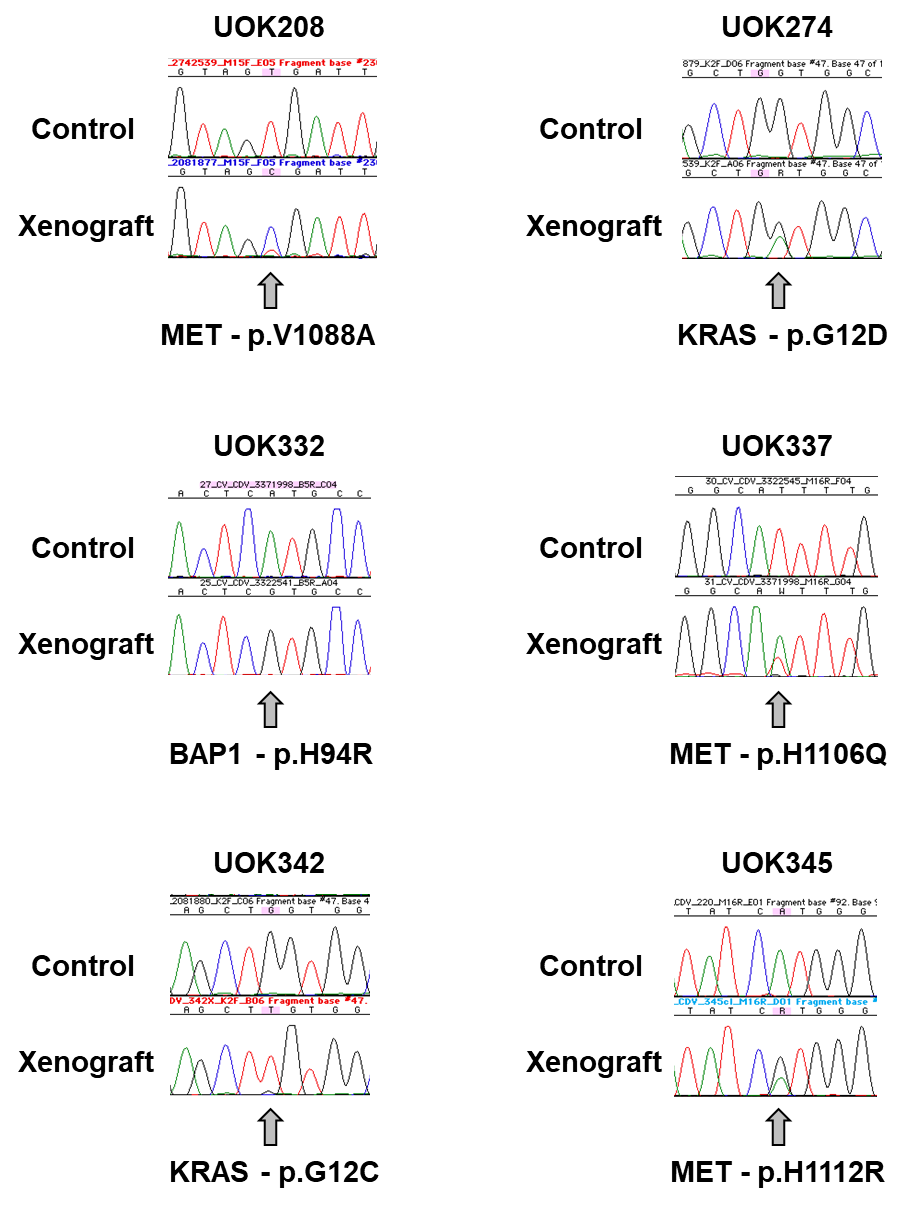


**Figure S8: Confirmation of type 1 PRCC cell line derived xenograft tumors**

A characteristic mutation was selected for each cell line and confirmed to be present in the xenografts produced from each respective cell line. All xenografts demonstrated the correct mutation upon analysis.


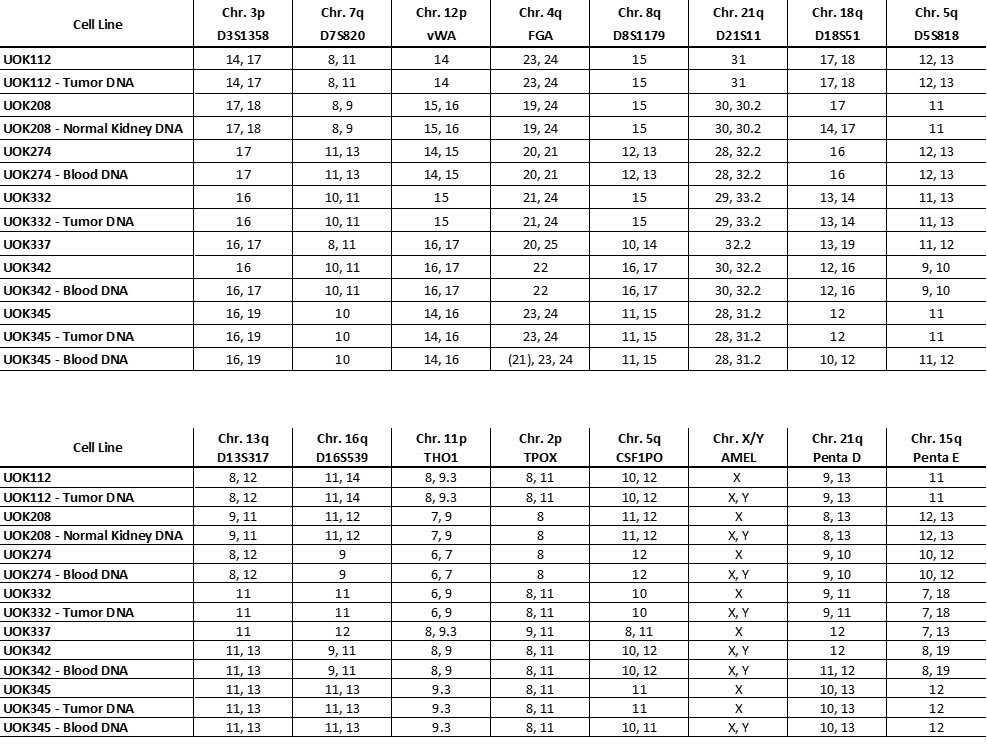


**Table S1: STR Profiles of type 1 pRCC cell lines and associated patient tissues**
